# Expanding role of deoxyribonucleic acid-sensing mechanism in the development of lifestyle-related diseases

**DOI:** 10.3389/fcvm.2022.881181

**Published:** 2022-09-13

**Authors:** Sachiko Nishimoto, Masataka Sata, Daiju Fukuda

**Affiliations:** ^1^Faculty of Clinical Nutrition and Dietetics, Konan Women’s University, Kobe, Japan; ^2^Department of Cardiovascular Medicine, Tokushima University Graduate School of Biomedical Sciences, Tokushima, Japan; ^3^Department of Cardiovascular Medicine, Osaka Metropolitan University, Osaka, Japan

**Keywords:** DNA-sensing mechanism, chronic inflammation, atherosclerosis, metabolic diseases, COPD, TLR9, STING, CKD

## Abstract

In lifestyle-related diseases, such as cardiovascular, metabolic, respiratory, and kidney diseases, chronic inflammation plays a causal role in their pathogenesis; however, underlying mechanisms of sterile chronic inflammation are not well-understood. Previous studies have confirmed the damage of cells in these organs in the presence of various risk factors such as diabetes, dyslipidemia, and cigarette smoking, releasing various endogenous ligands for pattern recognition receptors. These studies suggested that nucleic acids released from damaged tissues accumulate in these tissues, acting as an endogenous ligand. Undamaged DNA is an integral factor for the sustenance of life, whereas, DNA fragments, especially those from pathogens, are potent activators of the inflammatory response. Recent studies have indicated that inflammatory responses such as the production of type I interferon (IFN) induced by DNA-sensing mechanisms which contributes to self-defense system in innate immunity participates in the progression of inflammatory diseases by the recognition of nucleic acids derived from the host, including mitochondrial DNA (mtDNA). The body possesses several types of DNA sensors. Toll-like receptor 9 (TLR9) recognizes DNA fragments in the endosomes. In addition, the binding of DNA fragments in the cytosol activates cyclic guanosine monophosphate (GMP)-adenosine monophosphate (AMP) synthase (cGAS), resulting in the synthesis of the second messenger cyclic GMP-AMP (cGAMP). The binding of cGAMP to stimulator of interferon genes (STING) activates NF-κB and TBK-1 signaling and consequently the production of many inflammatory cytokines including IFNs. Numerous previous studies have demonstrated the role of DNA sensors in self-defense through the recognition of DNA fragments derived from pathogens. Beyond the canonical role of TLR9 and cGAS-STING, this review describes the role of these DNA-sensing mechanism in the inflammatory responses caused by endogenous DNA fragments, and in the pathogenesis of lifestyle-related diseases.

## Introduction

An organism needs to efficiently detect and resolve continual pathogenic attacks to maintain host-survival and homeostasis. The innate immune system protects the host from pathogenic infection by employing pattern recognition receptors (PRRs), which recognize pathogen-associated molecular patterns (PAMPs) and coordinate appropriate host defense mechanisms. PRRs include Toll-like receptors (TLRs), retinoic acid-inducible gene I-like receptors (RLRs), and NOD-like receptors (NLRs). After binding to their respective ligands, these receptors are activated, which results in the release of cytokines and chemokines. These first immune responses recruit antigen-presenting cells and leukocytes at the site of infection and induce subsequent adaptive immunity.

Toll-like receptors, which are the most familiar PRRs, are evolutionarily conserved and recognize various components came from bacteria, fungi, and viruses. There are 10 members of the TLR family in humans. Classically, most TLRs are categorized into two sub-groups. The first comprises TLR1, TLR2, TLR4, TLR5, TLR6, and TLR11 which are primarily expressed on the cell surface, and their function is to recognize the components of microbial membranes (1). The other sub-group is composed of TLR3, TLR7, TLR8, and TLR9. These TLRs are expressed intracellularly in vesicles [e.g., lysosomes, endosomes, and the endoplasmic reticulum (ER)] and recognize microbial nucleic acids (2–5). In the past, numerous studies have examined the downstream signaling related to TLRs and demonstrated that it requires the recruitment of several adaptor proteins, which lead to the activation of the nuclear factor-kappa B (NF-κB) and interferon (IFN) regulatory factor (IRF) pathways, accelerating inflammatory responses (2). In addition to TLRs which recognize nucleic acid, cytoplasmic DNA sensors have been known. In particular, stimulator of interferon genes (STING) which recognizes second messenger cyclic guanosine monophosphateâ adenosine monophosphate (GMP-AMP) (cGAMP) generated from cyclic GMP-AMP synthase (cGAS) activated by DNA fragments in the cytosol have been well-studied (6–9). RNA sensors in the cytosol, such as RLRs also have been known (10, 11). Numerous studies have reported that multiple pathways related to inflammation, such as IRFs, NF-κB, and inflammasomes, are activated after these DNA sensors bind to their ligands (12).

Non-communicable diseases (NCDs) are a major contributor to the global burden of disease and account for up to 72% of worldwide deaths (13). Chronic low-grade inflammation, characterized by persistent elevated concentrations of circulating pro-inflammatory cytokines, has been associated with the development of both age and diet-related NCDs, including obesity, cardiometabolic diseases, respiratory and auto-immune diseases, and many cancers (14–16). Recent studies have demonstrated that PRR signaling contributes not only to innate immune responses but also to the pathogenesis of various inflammatory diseases. Especially, the TLR9 signaling and STING signaling have attracted much attention, because emerging evidence suggested their roles in the pathogenesis of lifestyle-related diseases. Lifestyle-related diseases are a group of diseases that onset and progression closely link with lifestyle and behavior factor(s), such as dietary habits, physical activities, rest, smoking, alcohol consumption, etc. Especially cardiovascular diseases, metabolic disorders, respiratory diseases including chronic obstructive pulmonary disease (COPD), chronic kidney diseases (CKD) are focused as major lifestyle-related diseases, that are a health threat to humans in recent decades (17–19). Beyond the canonical role of TLR9 and cGAS-STING in antimicrobial and antiviral immunity, the functional roles of TLR9 and cGAS-STING to lifestyle-related diseases has emerged from recent expanding evidence. This review briefly summarizes the role of TLR9 and STING signaling in the pathogenesis of inflammation caused by self-derived DNA fragments. This review also highlights the roles of the DNA sensing system in the pathophysiology of lifestyle-related diseases and discusses its potential as a therapeutic target for these diseases.

## Deoxyribonucleic acid damage in lifestyle-related diseases

Sterile chronic inflammation is recognized as a shared mechanism of vascular diseases and metabolic diseases; however, the molecular mechanisms of sterile chronic inflammation remain a major medical problem that is yet to be solved. Though the mechanisms which cause DNA damage is multifactorial (20), DNA damage has been reported to play a crucial role in the development of these diseases (21, 22). Previous studies demonstrated that higher oxidative stress (23) and lower oxygen pressure (24) related to pathologic condition in unhealthy lifestyles and metabolic risk factors cause the deterioration of the cells in the vascular system and tissue of the metabolic organs. Subsequently, damaged genomic DNA and mitochondrial DNA (mtDNA) (25–29) are released and/or accumulated within the body (14, 30–32). We previously reported the accumulation of DNA fragments in macrophages, which infiltrate into atherosclerotic lesions and adipose tissue by using immune-electron microscopy and inflammatory activation of macrophages by DNA fragments (17–19). These results suggested that pro-inflammatory activation of macrophages by DNA damage play a key role in the pathophysiology of cardiometabolic diseases (33).

In developed countries, chronic kidney disease (CKD) is the most commonly attribute to diabetes and hypertension. The progression of CKD is associated with adverse clinical outcomes, including end-stage renal disease (ESRD), cardiovascular disease, and increased mortality (34–36). In the kidney, tubular cells contain enriched mitochondria to prepare for higher energy consumption. Recent studies highlight a pathogenic role of mitochondrial damage in the development of kidney disease. In fact, several kidney diseases such as diabetic nephropathy, tubulo-nephritis, and CKD show elevated mtDNA levels not only in the plasma but also in the urine (37–39). A recent study showed that urinary mtDNA levels have no significant association with the rate of worsening of renal function in non-diabetic CKD, although the levels correlate with baseline renal function, proteinuria, and the severity of histological damage (40).

Chronic obstructive pulmonary disease is a respiratory disorder characterized by irreversible limited expiratory airflow and abnormal inflammation. Etiologically, cigarette smoking (CS) is a major risk factor for COPD (41). Here, the impaired function of alveolar macrophages is a notable factor (42). CS-induced abnormal inflammatory responses amplify protease expression and oxidative stress, which accelerate COPD pathogenesis (43, 44). These processes further damage lung cells, including epithelial, vascular, and inflammatory cells, thus altering the lung microenvironment and enhancing the release of endogenous ligands. In fact, previous *in vitro* and *in vivo* studies have demonstrated that CS increased mtDNA damage (45, 46).

Deoxyribonucleic acid damage is also a potential marker of inflammatory diseases. The presence of extracellular DNA, which is named as cell-free DNA (cfDNA), has been known for a long time (47). Furthermore, recent studies have reported positive correlations between circulating cfDNA levels and the disease condition such as traumas (48–50), sepsis (51), cancer (52) or inflammatory diseases including autoimmune diseases (53–57), ESRD (58, 59), and neurodegenerative diseases (60). Recent clinical studies also have shown positive correlations between plasma cfDNA levels and the development of cardiometabolic disorders in humans. A clinical study that used coronary computed tomographic (CT) angiography demonstrated that patients with severe coronary artery disease had significantly higher levels of plasma double-stranded DNA and nucleosomes than those in the control group (61). Our previous study that used optical coherence tomography also revealed a positive correlation between cfDNA levels in the target artery and the inflammatory features of plaque in the target lesion of patients with acute myocardial infarction (17). In addition, we demonstrated that obese individuals presented higher cfDNA levels in the plasma and that the severity of abdominal adiposity and insulin resistance positively correlated with plasma levels of cfDNA (33). Similarly, several studies have reported that CS triggered DNA damage, releasing self-derived DNA into the plasma and alveolar space (62, 63). Thus, DNA damage and cfDNA has drawn increasing attention as a causal factor which initiates and accelerates vascular and metabolic diseases (20, 32).

Therefore, investigating pro-inflammatory roles of endogenous DNA fragments released from the host and the mechanisms by which endogenous DNA fragments accelerates inflammation associated with lifestyle-related diseases have become a research topic of great interest.

## Activation of toll-like receptor 9 and cyclic GMP-AMP synthase-stimulator of interferon genes

**FIGURE 1:**
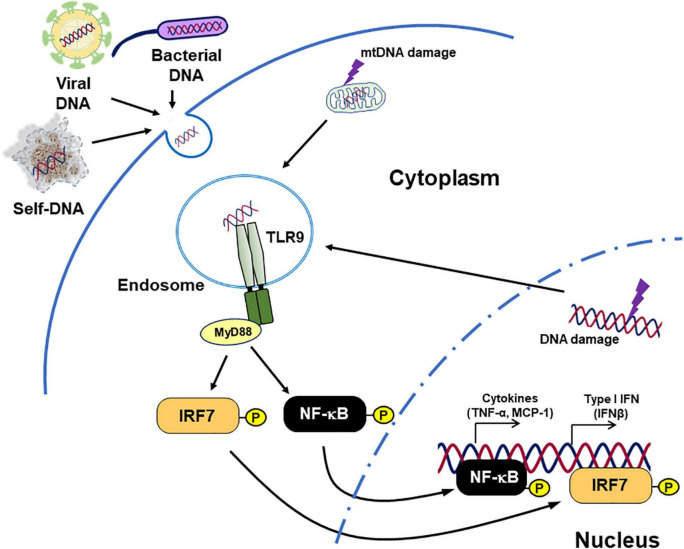
Inflammation caused by TLR9. TLR9 binds not only DNA originating from bacteria and viruses but also endogenous DNA and triggers signaling cascades related to pro-inflammatory responses. Diseases such as cancer, chronic infection, and lifestyle-related diseases can modulate TLR9 expression. In addition, tissue damage caused by these diseases increases the release of TLR9 agonists. This figure is reproduced from Fukuda et al. (80) with modification.

Toll-like receptor 9 is a well-studied DNA-sensing TLR. It recognizes unmethylated CpG motif-containing DNA fragments and induces innate immune response (64). After binding with ligands, TLR9 activates inflammatory pathways such as myeloid differentiation primary response 88 (MyD88)–IRF7 pathway and MyD88–NF-κB pathway, resulted in the production of type I IFN and inflammatory cytokines (Figure 1; 45, 65–67).

**FIGURE 2:**
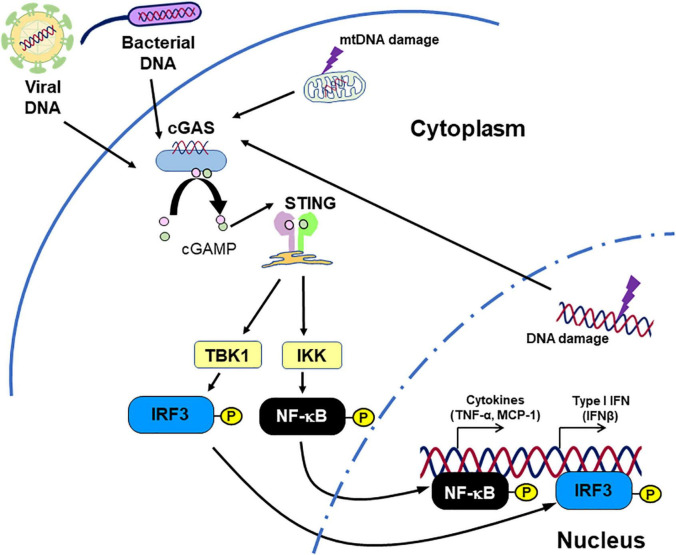
Inflammation caused by STING. STING recognizes cGAMP, which originates from DNA fragments. Not only DNA fragments derived from pathogens, but also endogenous DNA fragments can activate STING signaling, promoting inflammatory responses. This figure is reproduced from Fukuda et al. (80) with modification.

The cGAS-STING pathway is originally known as cytosolic DNA sensor machinery which recognizes pathogen-derived DNA, thus regulating the innate immune response (6, 68–70). STING ligates with cGAMP which is generated by cGAS activated with DNA fragments presented in the cytoplasm. Subsequently, STING activates NF-κB and IRF3, inducing IFNs and other pro-inflammatory cytokines (Figure 2; 7–9, 68, 71, 72).

## The role of deoxyribonucleic acid sensor in metabolic diseases

Due to lifestyle changes, the prevalence of obesity is increasing worldwide. This condition has close link with multiple metabolic abnormalities, including insulin resistance, and hepatic steatosis. Chronic sterile inflammation in metabolic organs plays a central role in the pathology of obesity and its associated complications. The mechanisms by which obesity promotes inflammation in metabolic organs are still undefined. However, obesity disturb the balance of the hypertrophy and the proliferation of adipocytes, angiogenesis, and the role of immune cells (24, 73, 74) because of higher oxidative stress, lower oxygen pressure (23), and excess inflammation, initiating cellular degeneration in adipose tissue (75–77). Here, local and/or systemic adipocyte-derived factors are suggested to participate in multiple pathways which accelerate inflammation within adipose tissue (77). Thus, many studies have been investigating the contribution of TLRs (78, 79). Recent studies, including our own, have demonstrated that among adipocyte-derived factors, self-derived DNA fragments released from metabolic organs promote chronic sterile inflammation by acting as endogenous ligands for DNA sensors.

We previously demonstrated that obesity caused by a high fat diet (HFD)-feeding increased plasma single-stranded DNA (ssDNA) levels in mice. Similarly, patients with visceral obesity, determined by CT, showed higher plasma ssDNA levels compared with the non-obese control. Furthermore, we reported that plasma ssDNA levels have a positive correlation with insulin resistance as determined by HOMA-IR, in humans (33). We also investigated the role of TLR9 in adipose tissue inflammation as it is one of the major receptors of nucleic acids (2). At first, we confirmed that HFD-induced obesity promotes TLR9 expression in the visceral fat. To explore the role of TLR9 in obese condition, we employed diet-induced obesity mouse model. TLR9 deficiency decreases macrophage accumulation into the visceral fat along with the reduction of inflammation in the adipose tissue and the inhibition of the development of obesity-induced insulin resistance (33). Similarly, pharmacological blocking of TR9 with iODN2088, a specific inhibitory oligonucleotide for TLR9, in HFD-fed wild-type mice suppressed inflammation in adipose tissue and ameliorated insulin sensitivity. In contrast, restoration of TLR9 only in BM aggravated insulin resistance in HFD-fed TLR9 deficient mice. In agreement with *in vivo* studies, our *in vitro* studies revealed that DNA fragments released from obese adipocytes partially promoted pro-inflammatory activation of macrophages *via* TLR9 signaling. Our results indicate that obesity overstimulates the innate immune system by increasing both ligand and receptor levels. In tandem, these results suggest a link between TLR9 and obesity-associated insulin resistance. Simultaneously, these results suggested that cfDNA-TLR9 signaling can be a potential therapeutic target for insulin resistance in obese subjects. Other TLR9 ligands, such as HMGB1, might also participate in TLR9 activation in obese subjects (81). However, a previous study reported opposite results. In that study, the authors demonstrated the protective role of TLR9 in the development of insulin resistance by showing the exacerbation of insulin resistance and pro-inflammatory activation of macrophages in TLR9 deficient mice (82). Further studies are needed to explore the function of TLR9 in the pathogenesis of obesity-induced insulin resistance.

Recent evidence has also demonstrated the contribution of TLR9 signaling to the pathogenesis of non-alcoholic fatty liver disease (NAFLD) (27). A preclinical study using a mouse model reported that TLR9 signaling activated by mtDNA accelerates the progression of hepatocyte damage and liver fibrosis, and that a TLR7/9 antagonist ameliorated the development of hepatic steatosis. As with animal studies, clinical studies have revealed that patients with non-alcoholic steatohepatitis (NASH) exhibits higher mtDNA levels compared to the controls (83, 84). Metabolic stresses caused by fat accumulation in the liver are thought to induce hepatocyte damage, leading to the release and accumulation of endogenous DNA fragments. DNA fragment-induced TLR9 activation might be an important driver of inflammatory responses in this widespread liver disease. Thus, TLR9 activation caused by self-derived DNA may be one of the molecular mechanisms of NAFLD.

In addition to TLR9, the expression of signaling molecules related to STING pathway is enhanced in metabolic organs in obese mouse models (85–87). For instance, the activation of cGAS– cGAMP–STING pathway by mtDNA derived from adipose tissues promoted chronic sterile inflammation in adipose tissue and contributed to the development of insulin resistance (87, 88). The activation of STING signaling in pancreatic β-cells was also documented in genetically obese mice such as db/db mice, suggesting that STING signaling is involved in the pathophysiology of type 2 diabetes (T2D) which is characterized by dysfunction of pancreatic β-cells (89).

Resembling TLR9, a number of studies have reported the role of STING signaling in the disease processes in NAFLD (90–92). The excess of fat accumulation in the liver triggers mitochondria dysfunction and mtDNA damage in hepatocytes (93). The activation of STING induced by these mtDNA fragments increases production of type I IFN (90, 91), accelerating oxidative stress and inflammation as hepatic diseases develop (94). In fact, genetically deficiency of STING ameliorated non-alcoholic steatohepatitis and insulin resistance in wild-type mice (90, 91). Here, Kupffer cells, a type of macrophage in the liver, are suggested to play a pivotal role. Kupffer cells increase the expression of inflammatory molecules such as TNF-α and interleukin-6 in the response to released mtDNA from hepatocytes, which accelerates disease processes (91). Furthermore, some clinical studies have shown potential contribution of STING signaling in patients with NAFLD by linking the release of mtDNA and the progression inflammation and fibrosis in the liver (92, 95).

Lifestyle and dietary changes tend to enlarge the number of patients with obesity and its complications worldwide. This could result in more attention being directed toward the role of TLR9 and STING signaling in the development of metabolic diseases, such as insulin resistance and hepatic diseases and advances being made in new therapeutic strategies. To explore the function of DNA sensors in the pathogenesis of metabolic diseases further studies are warranted.

## The role of deoxyribonucleic acid sensors in vascular diseases

Chronic inflammation in the vascular system initiates impairment of endothelial function, accelerating atherogenic process (96). Efficient intervention to risk factors such as dyslipidemia, T2D, and hypertension decreases cardiovascular events, although significant residual risk is still concerned (97). This also indicates that molecular and cellular mechanisms of atherogenesis are not completely understood.

Accumulating evidence demonstrates the participation of innate immune system in the process of vascular inflammation despite its multifactorial etiology (98). A variety of cells in arterial lesions, including endothelial cells, macrophages, and dendritic cells express PRRs, including TLRs (99–102). TLR9 plays a crucial role in atherosclerosis development. Several studies have demonstrated that the stimulation of TLR9 signaling accelerates pro-inflammatory activation of macrophages and dendritic cells (103–105). In addition, we found that ODN1826, a TLR9 agonist, partially through p38 MAPK signaling, increased the expression of pro-inflammatory molecules in apolipoprotein E-deficient (ApoE KO) macrophages (17). Previous studies have revealed the damage of vascular cells in atherosclerotic lesions (106–108), suggesting the release of various endogenous ligands for TLRs (109). In our *in vivo* study, genetic deletion of TLR9 suppressed atherogenesis in ApoE KO mice which received angiotensin II infusion (17). The blockade of TLR9 with the administration of iODN2088, an inhibitory oligodeoxynucleotide specific to TLR9, reduced atherosclerotic lesion development when compared to the control group in the same mouse model. Both genetical and pharmacological TLR9 blocking also abated the inflammatory features of atherosclerotic plaques at both the RNA and protein levels, while restoration of TLR9 in the bone marrow exacerbated atherogenesis in TLR9-deficient ApoE KO mice. These findings suggest that TLR9 has pro-atherogenic roles (17). Similarly, another study reported pharmacological blockade of TLR9 by IRS869 mitigated atherosclerotic lesion development and shifted macrophage polarization to the anti-inflammatory M2 phenotype (110). Pro-atherogenic properties of TLR9 was also reported by showing impaired reendothelialization and advanced atherosclerotic plaques in ApoE KO mice which received the administration of TLR9 agonist (111). Furthermore, we demonstrated that TLR9 contributes to the impairment of blood flow recovery in the ischemic limb by using a hind-limb ischemia model (18). TNF-α released from accumulated macrophages *via* TLR9 signaling played an important role. All these studies suggest that TLR9 activation enhances inflammatory responses and accelerates the development of vascular diseases.

Several groups have reported incongruous results by reporting that TLR9 has anti-atherogenic effects (112–114). Koulis et al. (114) demonstrated anti-atherogenic role of TLR9 by showing that TLR9-deficient ApoE KO mice exhibited increased inflammation in the plaque along with an increase in blood lipid levels. They also reported that the administration of CpG-ODN1668, a TLR9 agonist, suppressed atherosclerotic lesion development in ApoE KO mice. Thus, the role of TLR9 which have been reported is discrepant. Interestingly, a previous study reported conflicting roles of TLR9 activation depending on the concentration of its ligand (115). Therefore, the difference between the mouse model and experimental methods might result in the variance in the levels of ligands, which explains the discrepancy reported in previous studies. Further investigations are needed to clarify the effects of TLR9 on atherosclerosis.

A number of previous studies have investigated the role of cGAS-STING pathway as a major cytosolic DNA sensor and demonstrated its activation in response not only to pathogen-derived DNA but also endogenous DNA (6, 68–70). Amongst others, we have described the role of STING in the pathogenesis of atherosclerosis. To explore direct evidence of the contribution of STING signaling in vascular inflammation and subsequent atherogenesis, we first attempted to detect the presence of DNA damage in mouse atherosclerotic lesions by using WTD-fed ApoE KO mice, one of the widely used hypercholesterolemic mouse models, because released endogenous DNA initiates the production of STING ligands (19). The results of western blotting and immune-electron microscopy demonstrated the expression of γH2AX, a DNA damage marker, and the accumulation of DNA fragments in macrophages, respectively. Furthermore, we demonstrated the presence of cGAMP, a direct agonist of STING, in the atherosclerotic aorta of this mouse model using liquid chromatograph–mass spectrometry. Therefore, we deleted STING in ApoE KO mice to investigate its role in atherogenesis. Genetic deletion of STING decreased atherogenesis and attenuated the inflammatory features of the vasculature. Pharmacological blocking of STING using C-176 also decreased atherogenesis in the aorta compared to that in control groups. In contrast, its BM-specific expression promotes atherosclerotic lesion progression in ApoE KO mice. These results suggest causal roles of STING in the pathogenesis of atherosclerosis development. Additionally, a recent study showed that the genetic deficiency of IRF3, which is an adaptor molecule in downstream of STING signaling, attenuated the progression and the vulnerability of atherosclerotic plaques in ApoE KO mice (116). Similarly, administration of IFN-β, which is a downstream molecule of STING signaling, promotes atherogenesis in hypercholesterolemic mouse models (117). In our study, STING deficiency reduced the expression of IFN-β in the aorta of ApoE KO mice (19), which is consistent with these results. We further demonstrated that both cGAMP and mtDNA promoted the expression of inflammatory molecules, such as IFN-β, in both mouse and human macrophages (19). In addition, we demonstrated the expression of STING and cGAMP in atherosclerotic plaques collected by carotid endarterectomy, the levels of which were significantly higher in atherosclerotic lesions than control samples purchased from a tissue bank (19).

Recently, one study reported causal role of STING signaling in the pathogenesis of aortic disease (118). Genetic deletion of STING significantly decreased the aortic diameter, dissection, and aortic aneurysm formation in a mouse model. The underlying mechanisms included damage and release of DNA fragments from smooth muscle cells, and activation of macrophages by these DNA fragments. They also showed the inhibitory effects of C-176, a specific STING inhibitor, in their mouse model, suggesting the contribution of STING signaling to aortic aneurysm formation. The results of these studies suggest that the activation of STING signaling promotes vascular inflammation and that it could be a potential therapeutic focus for vascular diseases.

Contribution of STING to the development of vascular diseases has been also suggested in humans. The gain-of-function mutation in STING is reported to have close link with vasculopathy observed in STING-associated vasculopathy with onset in infancy (SAVI), which is a rare familial autoinflammatory disease (119–121). Enhanced IFN-β transcription in peripheral blood mononuclear cells in SAVI patients is thought to be one of the mechanisms involved (115). In contrast, one study reported protective effect of single-nucleotide polymorphism R293Q on STING on cardiovascular disease associated with obesity (122, 123). Evidence regarding the contribution of cGAS–cGAMP–STING signaling to vascular diseases is still limited; however, the results of recent studies suggest that STING signaling contributes to the pathogenesis of vascular diseases.

Thus, the role thought to be played by TLR9 and cGAS–cGAMP–STING signaling is expanding to vascular diseases in addition to the innate immune system. Further investigation of the role of these signaling in the development of vascular diseases would improve the understanding of the pathogenesis of atherosclerosis and might stimulate the development of new therapeutic approaches.

## The role of deoxyribonucleic acid sensor in kidney diseases

The roles of TLRs in inflammation observed in kidney diseases have been established in both animal models and patients. Several studies have confirmed the association between the TLR9 gene and CKD (124, 125). A human study demonstrated that patients with ESRD showed significant upregulation of TLR2 and TLR4, but not TLR7 or TLR9, in monocytes (126). On the other hand, polymorphisms of TLR9 have been confirmed to be associated with CKD in the Han Chinese population (124). A following study showed that the -1237T/C SNP of the TLR9 gene is significantly associated with ESRD in this population and that -1237T/C may be involved in the development of ESRD through transcriptional modulation of TLR9 (125). Therefore, TLR9 may play a critical role in the development of CKD.

Chronic kidney diseases is manifested by chronic inflammation, with continuous, unsuccessful injury-repair cycles and following fibrosis. These processes involve the activation of macrophages (127). Previous studies have reported the leading role of chronic inflammation and macrophage polarization in the progression of CKD. An animal study demonstrated that systemic exposure to CpG-DNA 1668, one of the TLR9 ligand, increases CD11b + /Ly6Chi macrophages and induces classically activated renal M1 macrophages that enhance intrarenal inflammation and disease progression of Alport nephropathy and other types of chronic kidney diseases (128). Activation of TLR9 induces accumulation of M1 macrophages and increased expression of pro-inflammatory cytokines in the renal interstitial compartment (129). Several studies also have explored the role of TLR9 using experimental acute kidney injury (AKI). Previous studies indicated that TLR9 does not contribute to the development of ischemic AKI by showing that TLR9 deficient mice were not protected against ischemic AKI (130, 131). In contrast, Han et al. reported that renal proximal tubular TLR9 activation exacerbates ischemic AKI by accelerating renal tubular inflammation, apoptosis as well as necrosis *via* NF-κB and caspase activation after ischemia-reperfusion injury, which leads to the development of AKI (132–134). The fibrosis of the kidney is another feature of AKI. One animal study using a mouse AKI-CKD transition model demonstrated that attenuation of CKD in the TLR9 deficient mice mainly relies on the effects of TLR9 on macrophages (129). In this study, TLR9 deficiency decreases the number of leukocyte and macrophage in the kidney following ischemia-reperfusion injury.

Chronic kidney diseases is associated with accelerated atherosclerosis progression and high incidence of cardiovascular events (135–138). An animal study using 5/6 nephrectomy or unilateral nephrectomy in ApoE KO mice demonstrated that CKD markedly accelerates atherogenesis in ApoE KO mice (136). They suggested that the CKD models which employed ApoE KO mouse are a useful tool to explore the mechanisms of uremic atherosclerosis. Due to a typical oxidative stress, CKD has emerged as a particularly strong risk factor for CVD (135). Thrombotic events are more likely to occur in patients with CKD, as well as in ApoE KO mice with CKD (138), suggesting that the plaques in CKD possess vulnerable features. A recent study using ApoE KO mice demonstrated that induction of CKD increases the release of mtDNA because of oxidative stress-induced mitochondrial damage, which activates the cGAS-STING pathway and subsequently induces type 1 IFN response in vascular smooth muscle cells (138). Interestingly, a recent study which used diabetic mouse models such as db/db mice and KKAy mice has reported that self-DNA-activated cGAS-STING pathway can be a new mechanism causing inflammation in the kidneys in diabetic condition (139). Alleviating type 1 IFN *via* cGAS-STING pathway may become a potential treatment strategy against diabetic kidneys as well as CKD-associated cardiovascular diseases.

These studies demonstrate that activation of the DNA sensors contributes to the development of kidney diseases, suggesting potential new therapeutic targets for preventing the progression of AKI, CKD, and diabetic kidney diseases. However, how DNA sensors affects CKD progression remains unclear. Further studies are needed.

## The role of deoxyribonucleic acid sensor in chronic obstructive pulmonary disease

Chronic obstructive pulmonary disease is a respiratory disorder characterized by irreversible limited expiratory airflow and aberrant inflammation. Inflammation plays a pivotal role in the pathogenesis of COPD (140, 141). The underlying molecular mechanisms are not completely understood; however, recent evidence has suggested that the innate immune system partially contributes to its pathogenesis (124, 142). Previous studies have determined the expression and function of TLRs in the development of COPD (143, 144).

The expression of TLR9 in the lungs (145) and its contribution to the pathogenesis of COPD have been reported (146). Foronjy et al. (147) demonstrated that genetic deletion of TLR9 prevents the development of CS-induced COPD in mice. The authors reported that in addition to inflammatory cells, epithelial cells play a pivotal role in COPD development. This is logical because the epithelium of the airways acts as the first line of defense against pathogens through the process of using a variety of receptors, such as TLRs. In immune responses, reactive-oxygen species (ROS) production is beneficial in the process of self-defense (148), and its accumulation is evident in patients with COPD (149). The overproduction of ROS is intended to abolish invading pathogens, meanwhile it can induce unwanted cellular damage. Excess ROS can directly initiate an inflammatory response and negatively affect tissue function and cellular structure (150). A previous study reported that ROS-induced mtDNA release stimulates immune cells because of TLR9 activation (151). The role and mechanism of activation of TLR9 in the development of COPD remain unclear; however, recent studies have reported that TLR9 polymorphisms are associated with both lung dysfunction and COPD (152, 153). Further study is required to understand the pathophysiology of COPD and develop novel therapeutic strategies that target TLR9.

Some viral-related innate immune mediators, such as RIG1, MDA5, LGP2, STING, and DAI are expressed in the lung tissue and bronchi of patients with COPD (154). Host-derived DNA fragments may act as pro-inflammatory signals for pulmonary inflammation (155). Recent data indicated the role of DAMPs in COPD (156) and many studies have attempted to reveal the relationship between STING and its ligand in the development of this particular disease (157). Nascimento et al. (62) showed that mouse CS-exposure promotes self-DNA release, which correlates with a neutrophil influx into the bronchoalveolar space *via* STING signaling. In a mouse model, acute CS exposure increased the self-DNA content in the alveolar space and accelerated the inflammatory response through the cGAS-STING pathway (62). Deslee et al. (158) demonstrated the nucleic-acid oxidation in alveolar fibroblasts of patients with severe emphysema. Similarly, CS exposure to mice accumulated nucleic acid oxidation in alveolar fibroblasts time dependently. DNase I treatment also reduced CS-induced lung inflammation (159). On the other hand, several previous studies have suggested that the suppression of cGAS-STING pathway and lower IFN levels are associated with a poor immune response to pathogens in patients with COPD (160, 161). These impeded STING-related immune responses may cause immune compromise in patients. Therefore, targeting the DNA-sensing mechanism such as STING is a double-edged sword. Further studies are needed to establish potential therapeutic strategies in lung diseases.

## Conclusion

**FIGURE 3:**
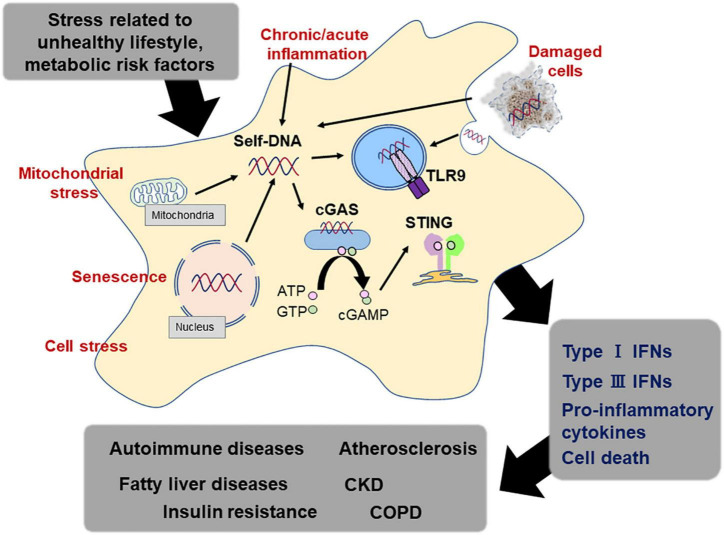
DNA sensing system and lifestyle-related diseases. Endogenous DNA fragments activate the DNA sensing mechanism, which participates in the activation of the immune response. Immune response caused by endogenous DNA fragments *via* DNA sensors such as TLR9 and STING accelerates sterile inflammation, leading to the development of lifestyle-related diseases, such as atherosclerosis, metabolic diseases, and pulmonary diseases.

The detection of exogenous DNA is the most fundamental function of the innate immune system and is the first line of self-defense in the human body (67). This system promotes inflammation against endogenous DNA fragments, as well as exogenous DNA, under certain circumstances. The underlying mechanism by which DNA-sensing mechanisms cause an unwanted immune response to host-derived DNA remains completely unknown. In this review, we synthesized findings from a very large and rapidly growing body of research investigating associations between DNA-sensing mechanism and the development of lifestyle-related diseases (80). The evidence suggests that host-derived DNA fragment including mtDNA potently works as a stimulator of TLR9 and/or cGAS-STING pathway under a certain circumstance, accelerating chronic inflammation (Figure 3). Although there are increasing number of studies, remaining discrepancies of the results may attribute to the study design such as experiment duration, types of agonists and antagonists, and animal background. Furthermore, methods of preparing DNA sample from blood or culture medium and quantification of the amount, sequences of DNA fragments which have higher potential as exogenous ligands for DNA sensors, and the origin of DNA fragments should be established to elucidate the importance of this system as a therapeutic target in the future.

As previous studies have demonstrated, risk factors, such as obesity, T2D, and dyslipidemia, induce tissue damage, which suggests the release of exogenous ligands, including nucleic acids. Therefore, controlling these risk factors by using the combination of medical treatment and a healthy lifestyle is indispensable in regulating inflammation caused by DNA-sensing mechanisms.

From the view of drug development, identifying the crosstalk between TLR9, cGAS-STING, and other types of DNA sensors of specific cell type in each stage of diseases will also help developing effective treatment in the future. Indeed, several animal studies have suggested that the administration of inhibitors for DNA sensors including TLR9 and STING attenuates the development of several lifestyle-related diseases such as insulin resistance, hepatic diseases, and atherosclerosis. However, DNA-sensing mechanism basically functions as self-defense. Further studies are needed to establish therapeutic strategies targeting this system. In addition, several studies have mentioned the crosstalk between TLR9 and cGAS-STING in response to DNA damage rerated with infectious diseases and some pathological conditions. In a mouse model of malarial infection, cGAS-STING pathway has been shown to induce suppressor of cytokine signaling (SOCS)1/3 to downregulate TLR9 signaling (162, 163). Deb et al. reported that triggering of the cGAS-STING pathway in plasmacytoid dendritic cells can induce expression of SOCS molecules, leading to inhibit the TLR9 pathway-mediated IFN production (162). In contrast, a synergistic role of TLR9 and STING by has also been reported in animal models such as acute peripheral tissue trauma models or other chemically induced lung injury (164, 165). Here, mtDNA activates neutrophils through both cGAS-STING and TLR9 pathways and leads to an increase in the production of neutrophil elastase and extracellular neutrophil-derived DNA in neutrophil extracellular traps, resulting in acceleration of subsequent sterile inflammation. At *in vitro* level, a study using cell lines such as human monocyte and human pDC has shown that a particular type of ODN can induce a strong cGAS-STING-dependent IFN response (166), suggesting that we need careful interpretation of the results derived from *in vitro* experiments which employed CpG-ODNs for distinguishing between TLR9- and cGAS-dependent effects. Until now, the number of studies which examined the crosstalk between multiple signal pathways related to innate immune systems in lifestyle-related diseases. Therefore, further detailed studies about the differences in the temporal and spatial expression and function of these innate immune signals in lifestyle-related diseases will help us to clarify the pathogenic roles of these DNA sensors in these diseases and provide evidence for further prevention and therapeutics.

In summary, DNA sensors, such as TLR9 and cGAS-STING, participate in the development of lifestyle-related diseases such as vascular, metabolic, kidney, and pulmonary diseases. These findings also highlight the potential benefits of transitioning to a healthy lifestyle to decrease disease risk. Further studies are required to understand the stimuli and mechanisms by which one of the most essential immune systems can play a harmful role, and to establish well-tolerated methods targeting DNA-sensing mechanism for these lifestyle-related diseases.

## Author contributions

All authors listed have made a substantial, direct, and intellectual contribution to the work, and approved it for publication.
